# Utility of key anatomical landmarks visualization using a radiopaque tape for successful extravascular implantable cardioverter defibrillator implantation

**DOI:** 10.1002/joa3.70112

**Published:** 2025-06-13

**Authors:** Satoshi Oka, Mitsuru Wada, Kohei Ishibashi, Nobuhiko Ueda, Kengo Kusano

**Affiliations:** ^1^ Department of Cardiovascular Medicine National Cerebral and Cardiovascular Center Suita Japan

**Keywords:** extravascular implantable cardioverter defibrillator, radiopaque tape

## Abstract

Radiopaque tape offers a practical and effective solution to visualize key anatomical landmarks for safe implantation of extravascular implantable cardioverter defibrillator (EV‐ICD).

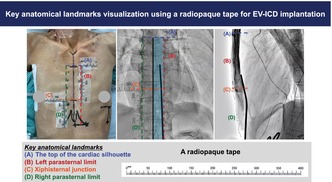

In addition to the traditional transvenous implantable cardioverter defibrillator (ICD) and subcutaneous ICD, a novel extravascular implantable cardioverter defibrillator (EV‐ICD, Aurora™, Medtronic, Inc., Minneapolis, USA) system is now available. The key feature of the EV‐ICD is its ability to deliver antitachycardia pacing (ATP) despite being an extravascular device.^1^

Substernal lead implantation enables ATP delivery alongside high R‐wave sensing capability.^2^ Although the feasibility of lead implantation in the anterior mediastinum has been demonstrated,^3^ careful procedure planning with attention to the key anatomical landmarks is essential to prevent complications such as organ injury and pleural placement. However, repetitive switching between anterior–posterior (AP) and left‐lateral (LL) views during substernal tunneling makes it challenging to maintain visibility of the key anatomical landmarks.

Although the placement of surgical instruments at each landmark maintains visibility, the instruments may move and interfere with the procedure. We propose an EV‐ICD implantation method using radiopaque tape (X‐measure™, Silcs Medical Inc., Fukushima, Japan) as a practical alternative solution to visualize key anatomical landmarks.

Herein, we present a case of a 75‐year‐old Japanese male patient with idiopathic dilated cardiomyopathy and ventricular tachycardia that was indicated for EV‐ICD implantation (Case 1, height: 163 cm, body weight: 40 kg, and body mass index: 15). Preoperative design lines and radiopaque tape placements are shown in Figure 1A. We covered the radiopaque tapes with a drape to strengthen their fixation after performing disinfection.

**FIGURE 1:**
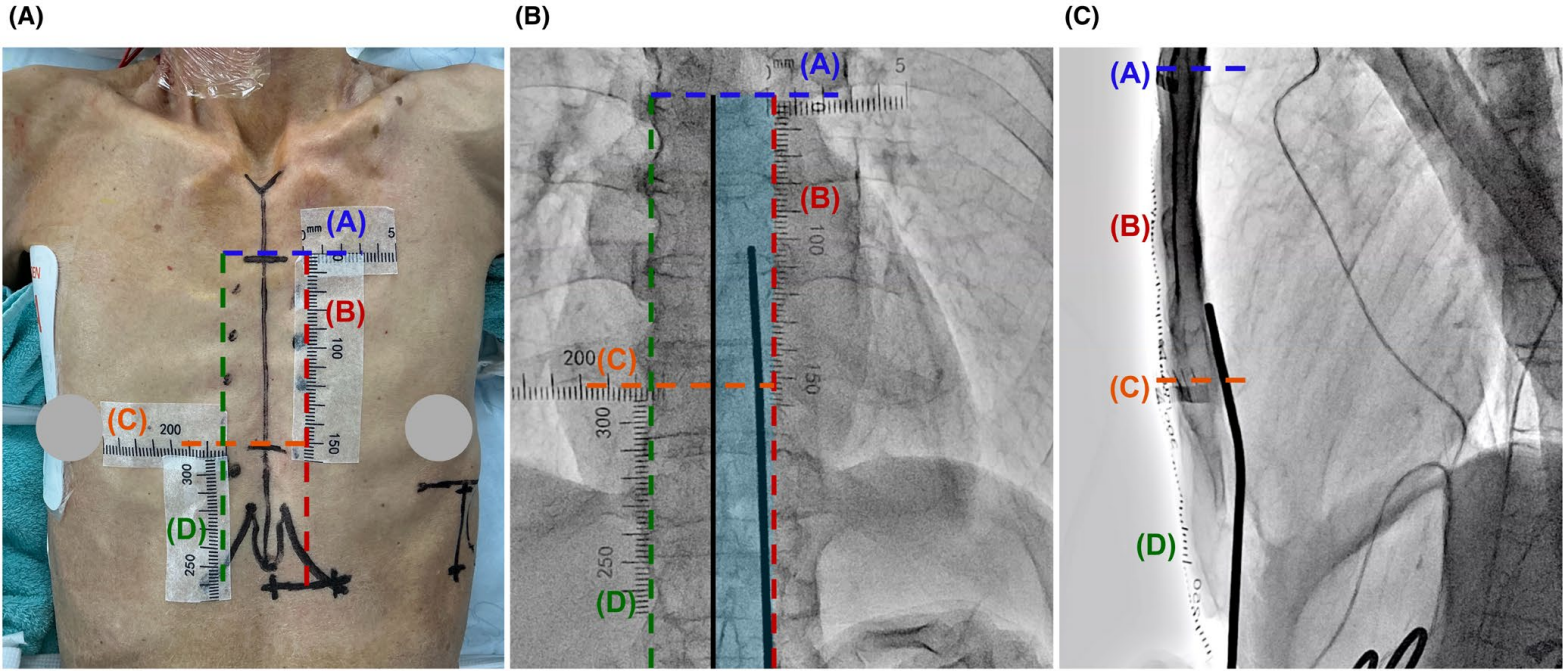
Radiopaque tape placements for visualizing key anatomical landmarks for successful extravascular implantable cardioverter defibrillator (EV‐ICD) implantation. (A) Preoperative design lines and radiopaque tape placements are shown from the anterior–posterior view. Tapes (A, B, C, and D) represent markers for the following anatomical landmarks: the top of the cardiac silhouette (blue dotted line), left parasternal limit (red dotted line), level of xiphisternal junction (orange dotted line), and right parasternal limit (green dotted line), respectively. Tape D is positioned below to measure the diameter from the anterior incision line to the xiphisternal junction. (B) Fluoroscopic image in the anterior–posterior view visualizing key anatomical landmarks with radiopaque tape. The safe substernal tunneling course (light blue zone) is defined by Tape A, Tape B, and the midline of the sternum (black line). (C) During the advancement of the substernal tunneling tool in the left‐lateral view, Tape A and Tape C highlight the upper limit of substernal lead implantation and the level of the xiphisternal junction, respectively.

Tape A marks the level of bronchial carina and the top of the cardiac silhouette, thereby acting as an upper limit marker for substernal lead implantation. Although this landmark is visible in the AP view (Figure 1B), it can be obscured in the LL view. However, the radiopaque tape ensured its visibility throughout the procedure (Figure 1C).

Tape B marks the left parasternal limit, thus preventing left internal thoracic artery injury and pleural placement (Figure 1A). Considering that the right‐sided and midline substernal implantations are associated with risks of P‐wave oversensing^3^ and cardiac injury, respectively, left‐sided substernal implantation is recommended. However, excessively left‐sided substernal and parasternal tunneling can lead to pleural complications. The use of radiopaque tape during the EV‐ICD implantation procedure facilitated careful substernal tunneling with clear visualization of the left parasternal limit in the AP view (Figure 1B).

Tape C marks the level of the xiphisternal junction, a key landmark for the target level of the ring 2 electrode (Figure 1), whereas Tape D indicates the right parasternal limit. The midline of the sternum was identified as the midpoint between Tape B and D in the AP view. Overall, the key anatomical landmark visualization using radiopaque tape effectively aided in safe EV‐ICD implantation.

We present an additional case of a 48‐year‐old Japanese female with hypertrophic cardiomyopathy who survived ventricular fibrillation (Case 2, height: 165 cm, body weight: 65 kg, and body mass index: 24). Owing to the breast thickness, the left breast was tensioned and fixed with a normal skin tape to the upper lateral side to reduce the displacement between the radiopaque tape and anatomical landmarks in the fluoroscopic image (Figure 2A).

**FIGURE 2:**
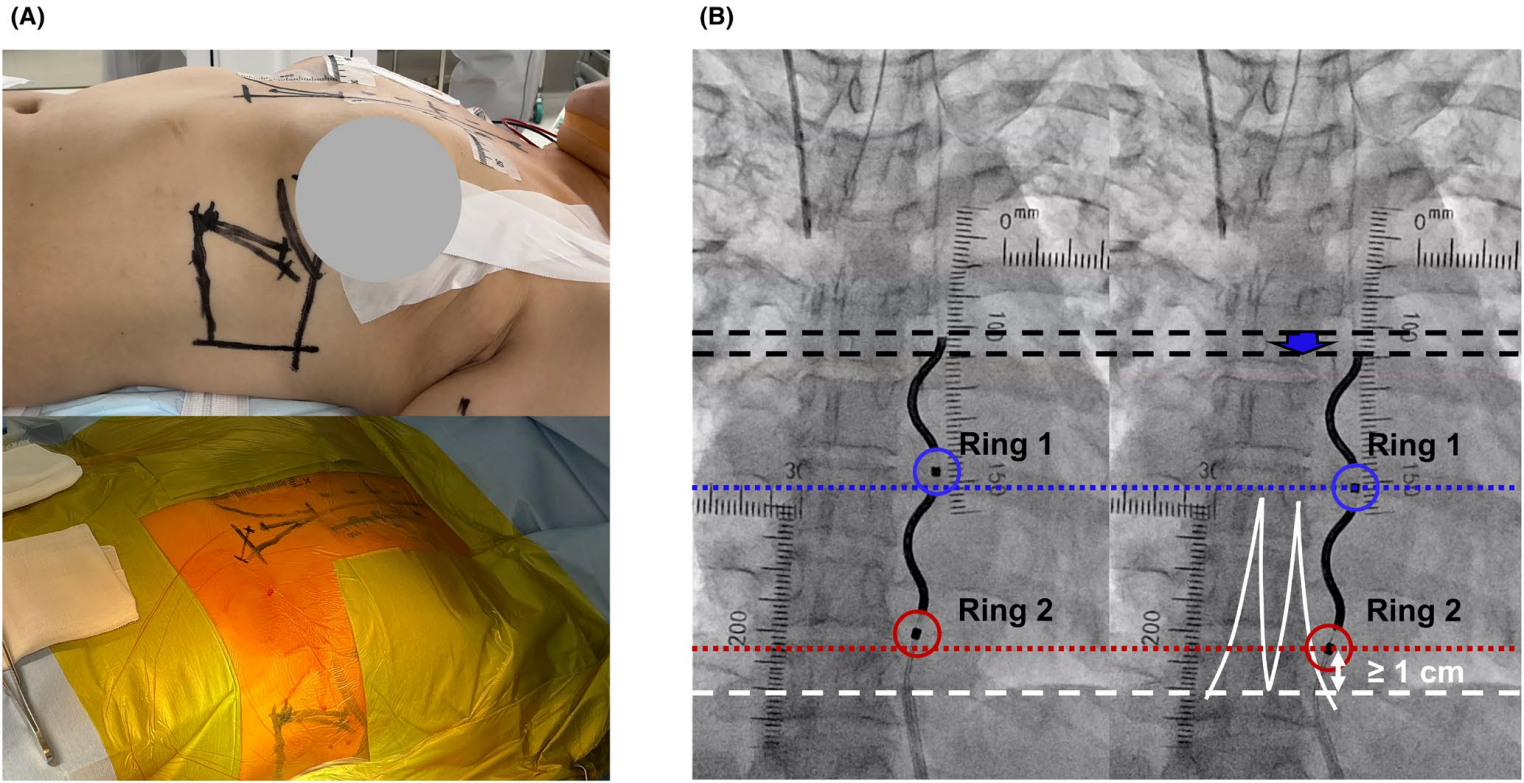
Radiopaque tape placements in a female case and additional utility of Tapes B and D. (A) Preoperative design lines and radiopaque tape placements in a female case are shown from the left‐lateral view. The left breast was tensioned and fixed with a normal skin tape to the upper lateral side to prevent mammary tissue injury because of the left‐lateral incision and to reduce the displacement between the radiopaque tape and anatomical landmarks in a fluoroscopic image because of the thickness of the breast. In addition, the radiopaque tapes were covered with a drape to strengthen their fixation after performing disinfection. (B) Tapes B and D are useful for identifying the differences in the levels of the substernal lead (black dotted lines) and electrodes (Ring 1, blue circle and dotted line; Ring 2, red circle and dotted line) between the baseline and the subsequent position after pulling the lead down for a repetitive sensing test. The estimated distance between ring 2 and the distal end of the xiphoid process can be measured using Tape D.

In this case, the sensing test initially failed at the upper lead position. However, an acceptable R‐wave amplitude was finally obtained without P‐wave oversensing after pulling the substernal lead slightly downwards (Figure 2B). Given that the sensing vector adopted in the sensing test is the near‐field vector between rings 1 and 2, the position of the ring electrodes is crucial for sensing test success. Tape B was useful for identifying the differences between the levels of the substernal lead and the ring electrodes before and after repositioning for a repetitive sensing test (Figure 2B). To prevent lead dislodgement, the ideal level of the ring 2 electrode is at least 1 cm above the xiphisternal junction. However, considering anatomical variations, the ring 2 position at least 1 cm above the distal end of the xiphoid process is judged acceptable. The estimated distance could be measured using Tape D, and the criteria for the location of ring 2 were easily judged (Figure 2B). Thus, this novel EV‐ICD implantation method using radiopaque tape was also feasible in a female case.

The EV‐ICD offers a useful alternative to traditional ICDs, with the added benefit of extravascular implantation and ATP delivery.^3^ This EV‐ICD implantation method that utilizes radiopaque tape has been employed in the initial six cases at our institution (between March 3 and April 2, 2025) without any procedure‐related complications (Figure 3).

**FIGURE 3:**
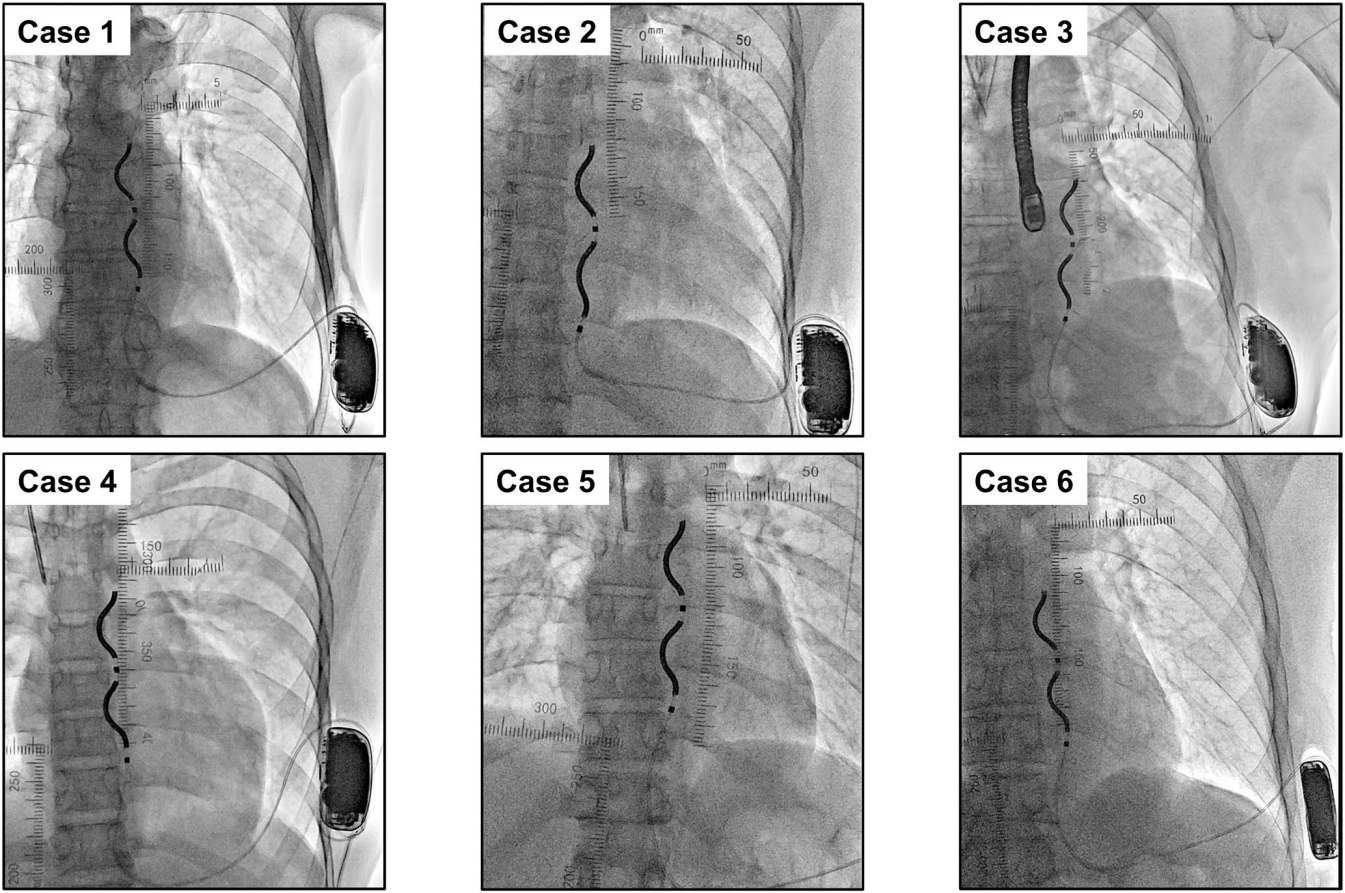
Fluoroscopic images of the initial six extravascular implantable cardioverter defibrillator (EV‐ICD) recipients obtained via anatomical landmarks visualization using radiopaque tape. Radiopaque tape placements and the final substernal lead position varied according to individual cases. Case 1: A 75‐year‐old male with idiopathic dilated cardiomyopathy. Case 2: A 48‐year‐old female with hypertrophic cardiomyopathy. Case 3: A 57‐year‐old male with arrhythmogenic right ventricular cardiomyopathy. Case 4: A 16‐year‐old male with hypertrophic cardiomyopathy. Case 5: A 53‐year‐old female with hypertrophic cardiomyopathy. Case 6: A 75‐year‐old male with ischemic cardiomyopathy.

However, this method has a possible limitation. Anatomical landmarks visualization using radiopaque tape may not be feasible in severe obesity cases owing to the thickness of the subcutaneous tissue. This will ultimately result in a mismatch between the radiopaque tape and anatomical landmarks in the fluoroscopic view. Although the normal skin tape traction of the breast may also be useful in obesity cases, we still have only a limited number of experiences.

Nonetheless, this case series with a small sample size showed that the thoracic anatomy and the final substernal lead position varied according to individual cases (Figure 3). Therefore, recognition of key anatomical landmarks is surely critical for successful EV‐ICD implantation.

Altogether, radiopaque tape offers a practical and effective solution to visualize key anatomical landmarks for safe implantation of EV‐ICD.

## CONFLICT OF INTEREST STATEMENT

Dr. Kengo Kusano, Dr. Kohei Ishibashi, Dr. Nobuhiko Ueda, and Dr. Satoshi Oka received remuneration for lectures from Medtronic Japan, Inc.

## APPROVAL OF THE RESEARCH PROTOCOL

M26‐150‐19.

## INFORMED CONSENT

The patient provided informed consent for the publication of this report and associated images.
